# MiR-155-5p suppresses SOX1 to promote proliferation of cholangiocarcinoma via RAF/MEK/ERK pathway

**DOI:** 10.1186/s12935-021-02374-0

**Published:** 2021-12-07

**Authors:** Da Wang, Fei Xiong, Guanhua Wu, Wenzheng Liu, Bing Wang, Yongjun Chen

**Affiliations:** 1 Department of Biliary-Pancreatic Surgery, Tongji Hospital, Tongji Medical College, Huazhong University of Science and Technology, Jiefang avenue 1095, Wuhan, Hubei China; 2 Department of General Surgery, The Fifth People’s Hospital of Shanghai, Fudan University, Shanghai, China

**Keywords:** Cholangiocarcinoma, ERK, miRNA, RAF, SOX1

## Abstract

**Background:**

Accumulating evidence has demonstrated the close relation of SOX1 with tumorigenesis and tumor progression. Upregulation of SOX1 was recently shown to suppress growth of human cancers. However, the expression and role of SOX1 in cholangiocarcinoma (CCA) is not well characterized.

**Methods:**

Expression levels of SOX1 in CCA tissues and normal bile duct tissues were examined using public GEO database. Western blot and immunohistochemistry were used to confirm the expression levels. Cell proliferation assay (CCK-8) and colony formation assay were performed to assess proliferation of CCA cells. A mouse model of subcutaneous transplantable tumors was used to evaluated proliferation of CCA in vivo. The putative regulating factor of SOX1 were determined using Targetscan and dual-luciferase reporter assay.

**Results:**

SOX1 was downregulated in CCA tissues. Overexpression of SOX1 significantly inhibited cell proliferation in vitro and suppressed tumor growth in vivo. miR-155-5p directly targeted the 3′-untranslated region (3′UTR) of SOX1 and inhibited expression of SOX1, resulting in the activation of RAF, MEK and ERK phosphorylation, and thus CCA proliferation. However, restoration of SOX1 expression in miR-155-5p overexpressing cell lines decreased the phosphorylation level of RAF, MEK and ERK, as well as the proliferation of CCA cells.

**Conclusion:**

MiR-155-5p decreased the expression of SOX1 by binding to its 3′UTR, which activated the RAF/MEK/ERK signaling pathway and promoted CCA progression.

**Supplementary Information:**

The online version contains supplementary material available at 10.1186/s12935-021-02374-0.

## Background

Cholangiocarcinoma (CCA) is a malignant tumor that originates from the epithelium of the bile duct. Patients with CCA have an extremely poor prognosis with 5-year survival rate of  < 10%. Surgery is the best curative treatment for CCA. However, CCA patients are often diagnosed at an advanced stage of the disease, and fewer than 30% patients present with surgically resectable disease [1–3]. Thus, for a vast majority of patients, chemotherapy is the only available therapeutic option, which has limited efficacy. Epidemiological studies have shown an increasing trend in the incidence of CCA over the past decades [4, 5]. Therefore, exploring the pathogenetic mechanisms of CCA and identification of novel biomarkers and therapeutic targets are key imperatives.

SOX (SRY-related HMG-box) domain proteins are a conserved group of transcriptional regulators characterized by the presence of a highly conserved high mobility group (HMG) domain that mediates DNA binding, and they are related to the mammalian testis determining factor gene sex-determining region Y (SRY) [6]. *SOX1* is one of the 20 *SOX* genes that have been identified in the mammalian genome. It has been shown to play an important role in embryonic development and regulation of stem cells [7, 8]. The SOX family is closely related to tumorigenesis and development; for example: SOX2 interacts with BCL11A to control the development of lung squamous cell carcinoma [9]; hypoxia may increase the hepatocellular carcinoma (HCC) stem cell population via altering the AR/miR-520f-3p/SOX9 signaling [10]; SOX17 is involved in the p53-mediated apoptosis pathway, and increases the sensitivity of endometrial cancer cells to cisplatin [11]. Studies have shown that SOX1 can inhibit the occurrence and development of HCC, esophageal cancer, nasopharyngeal carcinoma, lung cancer, and cervical cancer [7, 12–14]; however, its role in CCA has not been reported. Therefore, in this study, we explored the effect of SOX1 on CCA.

In this study, we found downregulation of SOX1 in CCA. Overexpression of SOX1 inhibited the proliferation of CCA, both in vitro and in vivo. MiR-155-5p inhibited SOX1 expression by combining with the 3′-untranslated region (3′UTR) of SOX1, and activate the RAF/MEK/ERK pathway to promote CCA progression. Our findings demonstrate the critical role of miR-155-5p/SOX1/RAF/MEK/ERK axis in CCA progression, which may provide a novel diagnostic and therapeutic target for CCA.

## Methods

### Cell lines, lentivirus infection and miRNA transfection

Human CCA cell line TFK-1 was purchased from DSMZ (Deutsche Sammlung von Mikroorganismen und Zellkulturen GmbH, Braunschweig, Germany). TFK-1 derives from primary partly-differentiated tubular adenocarcinoma from common bile duct. HUCCT-1 cell line was purchased from the RIKEN bioresource Center (Saitama-ken, Japan). HUCCT-1 derives from primary moderately-differentiated adenocarcinoma from intrahepatic bile duct. TFK-1 and HUCCT-1 cells were cultured in RPMI 1640 supplemented with 10% fetal bovine serum (FBS) in a humidified incubator containing 5% CO_2_ at 37 °C. HEK293T cells were cultured in DMEM supplemented with 10% FBS.

Lentivirus for overexpressing SOX1 (LV-SOX1), ERK (LV-ERK), downregulation SOX1 (SOX1-KD), negative control lentivirus of SOX1 (NC-SOX1) and negative control lentivirus of ERK (NC-ERK) were purchased from Genechem (Shanghai, China). Cells were infected with lentivirus particles for 24 h, and then cultured in fresh medium. The MOI of lentivirus to TFK-1 and HUCCT-1 is 20. Stable cell lines were generated via puromycin (5 μg/mL) selection.

All miRNA oligos were purchased from Guangzhou RiboBio:

miR-200b-3p: UAAUACUGCCUGGUAAUGAUGA; miR-155-5p: UUAAUGCUAAUCGUGAUAGGGGUU; miR-144-3p: UACAGUAUAGAUGAUGUACU; miR-NC: UUUGUACUACACAAAAGUACUG. miRNA transfection was performed using Lipofectamine 2000 (Invitrogen). Firstly, the cells to be transfected were inoculated with 1 × 10^5^ cells in a six-hole plate, add 2 ml medium, culture overnight in 37 ℃ incubator. Next day, added 5 μl lip2000 (Intritrogen) to 250 μl OPTIM medium, then added 2 μl miRNA (20 μM, all the miRNA in this article are purchased from Guangzhou RiboBio) to 250 μl OPTIM medium (plasmid for 2 μg), mixed gently at room temperature. After 5 min, mixed miRNA medium with Lip2000 medium for 20 min at room temperature. Finally, add these medium to the cells prepared yesterday and supplement the RMPI1640 medium to 1 ml, shake well, place in 5% CO_2_ at 37 °C incubator for 6 h. After 6 h, change the medium to RMPI1640. After 72 h, protein can be extracted for identification.

### Immunohistochemistry

Nine CCA specimens and four normal bile duct tissue specimens were obtained from the Tongji Hospital of Huazhong University of Science and Technology (HUST). The clinicopathologic characteristics of patient was showed in Table 1. The tissue samples were obtained with the approval by the Ethical Committee of HUST and after obtaining written informed consent of the patients. The samples were formalin-fixed, paraffin-embedded on slides, and characterized according to standard pathology. Immunohistochemistry was performed on 3-μm serial sections as described previously [15]. In brief, the sections were subjected to deparaffinization and hydration steps followed by quenching and peroxidase reaction steps. Antigen retrieval was performed by microwave irradiation for 8 min in citric acid (0.01 M). Following one hour of blocking in 10% goat serum, tissue sections were immunostained with SOX1 antibody (1:200), incubated with secondary antibody (Elivision ^TM^super HRP (Mouse/Rabbit) IHC Kit, KIT-9922, Biotechnologies) for 10 min, followed by 3,3-Diaminobenzidine tetrahydrochloride (DAB) staining, and then counterstained with hematoxylin. Immunohistochemical signals were scored semi-quantitatively, according to the percentage and intensity of positive-staining cells. 0: < 5% positive cells; 1: 5–24% positive cells; 2: 25–49% positive cells; 3: 50–74% positive cells and 4: ≥ 75% positive cells. Intensity was scored as 0 for absence of staining, 1 for weak, 2 for moderate, and 3 for strong staining. The final staining index was equal to intensity  ×  positive rate and classified as absent, 0–1; mild, 2–4; moderate, 5–8; and strong, 9–12 respectively.

**Table 1:** Association between SOX1 expression and clinicopathologic characteristics

Clinicopathologic characteristics				
Clinical charactics	Numbers of patients	SOX1		P value
		Low expression	High expression	
Age (years)				0.405
≤ 60	4	2	2	
> 60	5	4	1	
Gender				0.405
Male	5	4	1	
Female	4	2	2	
Tumor size (cm)				0.048
≤ 3	4	1	3	
> 3	5	5	0	
Lymph node metastasis				0.226
Negative	6	5	1	
Positive	3	1	2	
TNM stage				0.405
I and II	5	4	1	
III and IV	4	2	2	

Low expression: IHC staining index  ≤  6; high expression: IHC staining index  > 6

### Bioinformatics analysis

Three independent CCA gene expression profiles GSE32225 (which includes 149 cholangiocarcinoma samples and 6 normal biliary epithelial cell samples) [16], GSE76297 (which includes 91 cholangiocarcinoma samples and 92 non-tumor tissue samples) [17], and GSE32957 (which includes 16 cholangiocarcinoma samples and 5 non-tumor tissue samples) [18] were obtained from the Gene Expression Omnibus database (GEO). GSE32225 is based on the platform Illumina HumanRef-8 WG-DASL v3.0 (GPL8432), GSE76297 is based on the platform Affymetrix Human Transcriptome Array 2.0 (GPL17586) and GSE32957 is based on the platform Nanostring nCounter Human microRNA Expression (GPL14732). All the data was normalized according to the description and available freely online. Differences between groups of patients were assessed with one-way analysis of variance (ANOVA) using Graphpad Prism (version 7.0, San Diego, CA).

### Western blot analysis

Cells were lysed in RIPA lysis buffer (50 mM Tris–HCl, 150 mM NaCl, 1 mM EDTA-Na2, 1% Triton X-100, 1% sodium deoxycholate, 0.1% SDS) supplemented with protease inhibitors and phosphatase inhibitor on ice for 15 min. The cell lysates were cleared by centrifugation at 12,000×*g* for 10 min, then heat-denatured with 5 ×  Laemmli buffer (G2013, Servicebio). The prepared samples with equal amount of total protein (20 μg) were subjected to SDS-PAGE and immunoblotting, as per the standard procedure. The following antibodies were used: anti-SOX1 (ab109290, Abcam), anti-β-catenin (ab32572, Abcam), anti-HES1 (#11988, Cell Signaling Technology [CST]), anti-PROX1 (11067-2-AP, Proteintech), anti-p38 (#8690, CST), anti-phospho-p38 (#9215, CST), anti-ERK (#4695, CST), anti-phospho-ERK (#4370, CST), anti-AKT (#4691, CST), anti-phospho-AKT (#4060, CST), anti-JNK (#9252, CST), anti-phospho-JNK (#700031, Invitrogen), anti-RAF (#9422, CST), anti-phospho-RAF (#9427, CST), anti-MEK (51080-1-AP, Proteintech), anti-phospho-MEK (#9154, CST), anti-α-Tubulin (11224-1-AP, Proteintech), anti-GAPDH (BM1623, Boster), anti-BCL2 (Proteintech, 12789-1-AP), anti-PCNA (BM0104, Boster). All antibodies were diluted by antibody dilutions (G2025, Servicebio) with a ratio of 1:1,000. All quantitative analyses were performed using the software image J.

### RNA extraction and quantitative real-time PCR (qRT-PCR)

Tissue samples were subjected to total RNA isolation using the RNA isolater Total RNA Extraction Reagent (Vazyme, Nanjing, China) and reverse-transcribed into cDNA using the HiScript III RT SuperMix for qPCR (+  gDNA wiper) (Vazyme) according to the manufacturer’s instructions. qPCR was then performed using the ChamQ Universal SYBR qPCR Master Mix (Vazyme) in the iQ5 quantitative PCR detection system (Bio-Rad, Richmond, CA,USA). The extraction, reverse and qPCR of miRNA were using a miRcute kit (TIANGEN BIOTECH, Beijing) in accordance with the manufacturer’s introductions. We used 2^−ΔΔCt^ method to analysis the relative expression of genes. The primer sequence of the genes were listed below: SOX1, Forward (F): 5′-AGACCTAGATGCCAACAATTGG-3′, Reverse (R): 5′-GCACCACTACGACTTAGTCCG-3′; GAPDH, F: 5′-GGAGCGAGATCCCTCCAAAAT-3′, R: 5′-GGCTGTTGTCATACTTCTCATGG-3′; U6, F: 5′-CTCGCTTCGGCAGCACA-3′, R: 5′-ATCCAGTGCAGGGTCCGAGG-3′; hsa-miR-155-5p, F: 5′-CGCGTTAATGCTAATCGTGATAGGGGTT-3′, R: 5′-ATCCAGTGCAGGGTCCGAGG-3′.

### Cell proliferation assay and colony formation assay

The different groups of cells (2 × 10^3^ cells/well) were cultured in 96-well plates in 10% FBS RPMI 1640 medium. During the last 1 h culture, individual wells were added with Cell Counting Kit-8 (CCK-8) reagent (Dojindo, Tokyo, Japan). The cell proliferation was measured for the optical density (OD) at 450 nm using a microplate reader (Bio-Rad). For colony formation assay, cells were cultured in 3-cm dishes for 2 weeks, fixed with 4% paraformaldehyde for 30 min, then stained with 1% crystal violet for 30 min. Colonies with diameter greater than 100 μm were counted.

### Apoptosis

Annexin V-APC/7-AAD apoptosis kit (AT105, MULTI SCIENCES, China) was used to detect cell apoptosis according to the manufacturer’s instructions. In brief, 2 × 10^6^ cells were washed with PBS, then added 500 μl Apoptosis Positive Control Solution to rehang the cells, place the tube on ice for 30 min. Resuspended in 1 ×  binding buffer with annexin V-APC (5 μl per tube) and 7-AAD (10 μl per tube) and incubated in the dark for about 5 min. Finally, cells were analyzed by flow cytometry.

### Xenografts

Male BALB/C nude mice (age: 4 weeks) were obtained from the Beijing Vital River Laboratory Animal Technology, Beijing, China and housed in a pathogen-free facility. For mouse xenograft models, TFK-1 and HUCCT-1 cells (2 × 10^5^ per mouse) expressing either SOX1 or negative control vector in 200 µL PBS were implanted subcutaneously into the right flank of the mice. We divided the mice into four groups (5 mice per group) to injected the cells above respectively. Mice were sacrificed 18 days post-inoculation. All mice had tumors developed in their right flank. Tumor masses were measured with a digital Vernier caliper and tumor volume (in cubic millimeter) calculated using the formula: width^2^ ×  length/2. The protocols for the animal study were approved by the Experimental Animal Ethics Committee of the Tongji Medical College of Huazhong University of Science and Technology.

### Luciferase reporter activity assay

The psiCHECK-2 luciferase reporter vector was purchased from Promega. PCR-mediated site-directed mutagenesis was used to generate mutation as indicated. The SOX1-WT-3’UTR and SOX1-Mutant-3′UTR luciferase reporter constructs were generated by inserting either wildtype or mutant 400 bp fragments of the predicted 3’UTR region of human *SOX1* gene into the XhoI and NOTI multi-clonal sites of psiCHECK-2 vector.

The sequences of inserted 3′UTR region are listed below:

SOX1-WT, 5′-GCTAACCGATGTGAACGCAAAATGCCTTGTTCATTATTCCTGACGAGATCTTGAGGTTGTTTGATGCTTTAAATTTTTTAATTATATTATTTTCTAGGTGTTTATTGGTACATTGCAGTTTTTTTTTTGAAATTTAAAAATTTCTGTAAAACTTTGTCTTCAAGTAATCTGACAGCATTAAATATTGCATTTAAAAATTATACTGTAGCAAATACATTTAAAAATTAATCACAACGTTAAGATGAAATTATATTTTTGGAAAAAAAAAACACTTGAAGCCCAGATGGAAATACGTTTATTTCAGCAGCCTTAGGTTTCCCCTCGCTTTCTCAACACCCTTCCTTGTCCTGGAGTATGGACTGTCCGTCCAAAAGTGAGCCTATGCTATAAGTTTAATGAG-3′; SOX1-mut, 5′-GCTAACCGATGTGAACGCAAAATGCCTTGTTCATTATTCCTGACGAGATCTTGAGGTTGTTTGATGCTTTAAATTTTTTAATTATATTATTTTCTAGGTGTTTATTGGTACATTGCAGTTTTTTTTTTGAAATTTAAAAATTTCTGTAAAACTTTGTCTTCAAGTAATCTGACTCGTAATTATATTGCATTTAAAAATTATACTGTAGCAAATACATTTAAAAATTAATCACAACGTTAAGATGAAATTATATTTTTGGAAAAAAAAAACACTTGAAGCCCAGATGGAAATACGTTTATTTCAGCAGCCTTAGGTTTCCCCTCGCTTTCTCAACACCCTTCCTTGTCCTGGAGTATGGACTGTCCGTCCAAAAGTGAGCCTATGCTATAAGTTTAATGAG-3′.

HEK293T cells were co-transfected with the indicated plasmids and miR-155-5p or miR-NC for 48 h. Firefly and Renilla luciferase activities were measured using the dual luciferase reporter assay system (Promega), according to the manufacturer’s protocol.

### Statistical analysis

All data are expressed as mean  ±  S.D. Differences between two independent samples were assessed using two-tailed Student’s *t* test and differences among the groups were determined by two-tailed ANOVA using GraphPad Prism 5.0 (GraphPad Prism Software, San Diego, CA, USA). P values  < 0.05 were considered indicative of statistical significance.

## Results

### SOX1 is down-regulated in CCA tissues

**Fig. 1:**
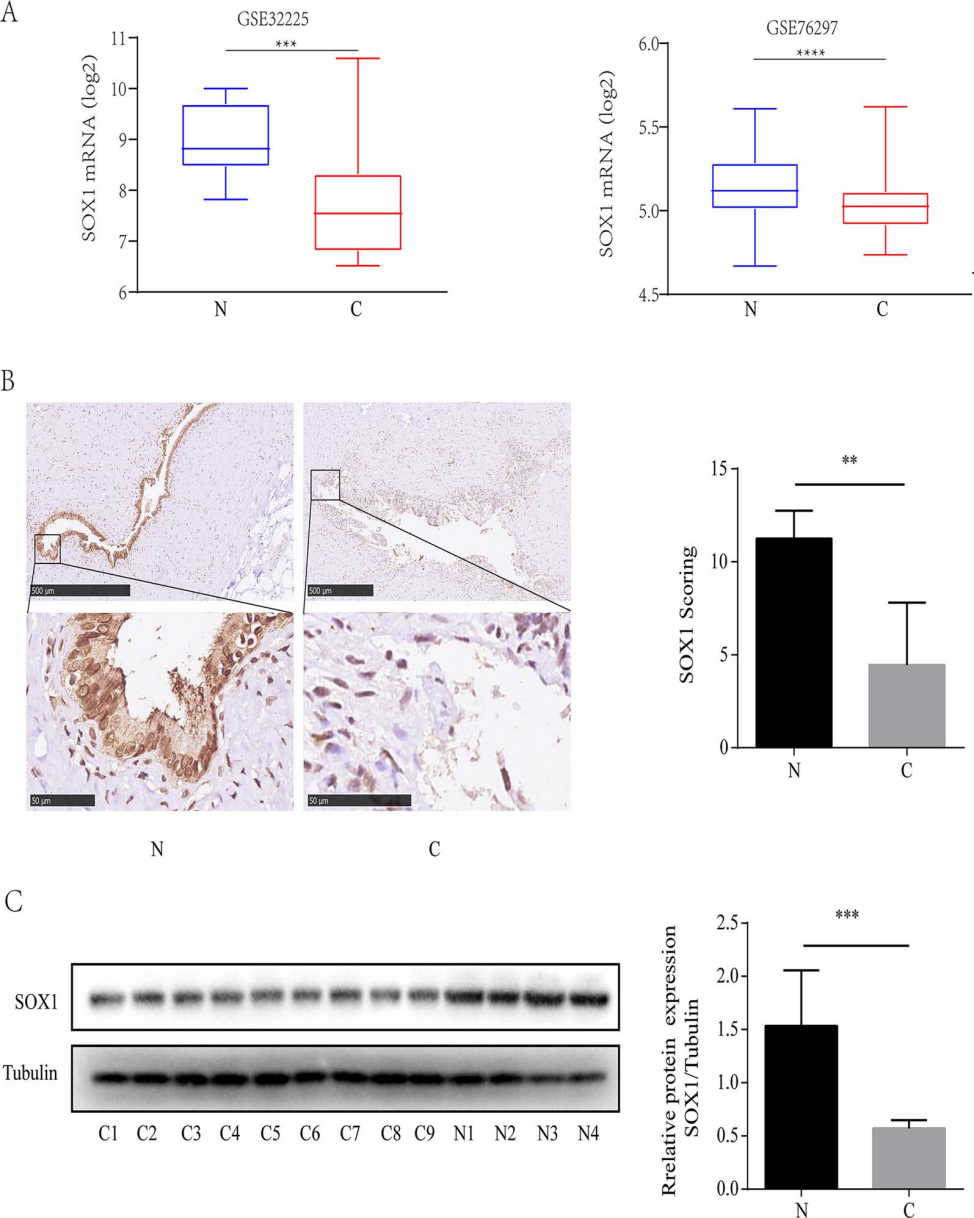
SOX1 is down-regulated in CCA tissues. **A** Box plots of SOX1 expression in cholangiocarcinoma (CCA) tissues compared to normal bile duct tissues. Data sourced from GEO databases GSE32225 (**A**) and GSE76297 (**B**). ***p  < 0.001, ****p  < 0.0001. **B** Representative IHC images showing SOX1 expression in normal bile duct tissue and CCA tissue specimens. Upper panel shows the overall view of the entire section. Scale bars are shown. The SOX1 expression were examined by semiquantitatively analyzed. **p  < 0.01. **C** Results of Western blotting showing the expression levels of SOX1 protein in nine CCA tissue specimens and four normal bile duct tissue specimens. *N* normal bile duct tissues; *C* CCA tissues

To investigate the functions of SOX1 in CCA, we first examined the expression of SOX1 in CCA patients based on data from the public GEO database (GSE32225, GSE76297). We found downregulation of SOX1 in CCA compared with the normal bile duct tissues (Fig. 1A). Subsequently, we detected the protein expressions of SOX1 in 13 clinical samples (9 CCA and 4 normal bile duct tissue specimens) using immunohistochemistry and Western blot analysis, and verified the results from the public database. The results showed significant downregulation of SOX1 protein expression in primary CCAs compared with normal bile duct tissues (Fig. 1B, C), which were consistent with the above results. Next, we explored how the expression of SOX1 was associated with CCA. We found that low level of SOX1 was associated with tumor size (P  < 0.05), but not with age, gender, lymph node metastasis and TNM stage (Table 1), which indicate that SOX1 may associated with proliferation of tumor.

### SOX1 inhibits CCA cell proliferation in vitro and suppresses tumor growth in vivo

**Fig. 2:**
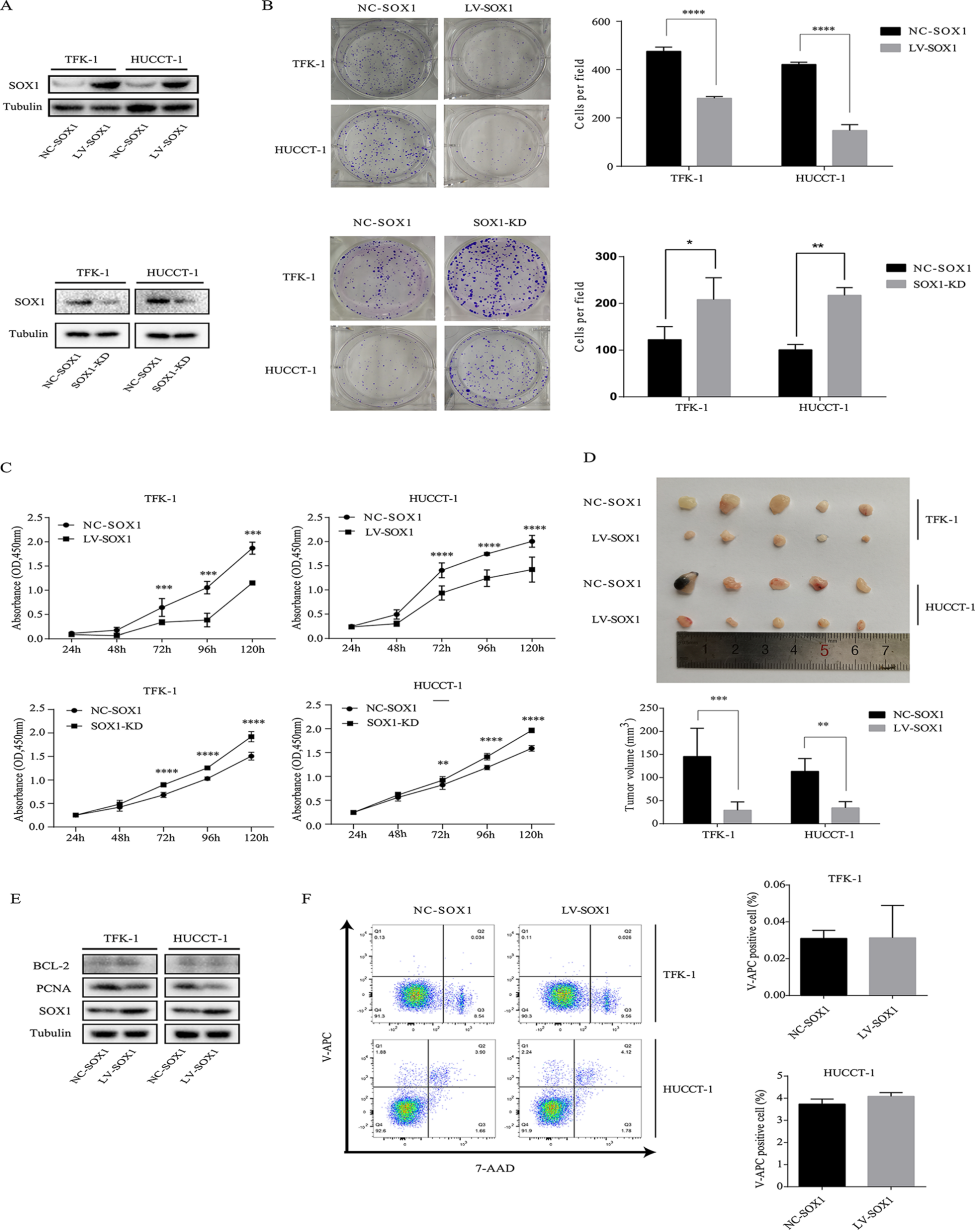
SOX1 inhibits CCA cell proliferation in vitro and suppresses tumor growth in vivo. **A** TFK-1 and HUCCT-1 cells were transfected with NC-SOX1, LV-SOX1 and SOX1-KD for 72 h. SOX1 protein level was assessed by Western blotting. **B** Representative images of colony formation assay (left panel) and analysis of colony numbers (right panel). *p  < 0.05, **p  < 0.01, ****p  < 0.0001. **C** Cell proliferation was assessed by CCK-8 assay. ***p  < 0.001, ****p  < 0.0001. **D** Above panel: Xenograft tumors at day 18 after implantation of NC-SOX1 or LV-SOX1 cells into the right flank of nude mice. Below panel: comparison of tumor volumes between NC-SOX1 and LV-SOX1 xenograft mice. **p  < 0.01, ***p  < 0.001. **E** Protein of xenograft tumors was extract and PCNA, BCL2, SOX1 was assessed by Western blotting. **F** Flow cytometry detected apoptotic cells after cells were transfected with LV-SOX1 and NC-SOX1

Since rapid growth of tumor is the main cause of poor prognosis, we investigated the impact of SOX1 on the growth of CCA. We induced overexpression and downregulation of SOX1 in TFK-1 and HUCCT-1 cells through lentiviral-mediated transfection carrying SOX1 (LV-SOX1) and SOX1-RNAi (SOX1-Knock Down, SOX1-KD) to assess the effect of cell proliferation. Western blot was performed to confirm the stable overexpression and downregulation of SOX1 in TFK-1 and HUCCT-1 cells (Fig. 2A). Then, the effect of SOX1 on cell proliferation was assessed using plate clone assay and CCK-8 assay. As shown in Fig. 2B, C, CCA cells with LV-SOX1 showed lower proliferation rate and formed fewer colonies than NC-SOX1. The subcutaneous tumor growth of TFK-1 and HUCCT-1 cells with LV-SOX1 and NC-SOX1 transfected in BALB/C nude mice is shown in Fig. 2D. The tumor volume in mice injected with LV-SOX1 CCA cells were significantly smaller than the NC-SOX1 group. To explore the tumor growth inhibitory effect of SOX1, BCL-2 and PCNA was detected by western blot of xenograft tumor samples. Results showed PCNA was downregulated in LV-SOX1, with no significant change in BCL-2 (Fig. 2E). The quantification of western blots of Fig. 2E is showed in Additional file 1: Figure. S1A. To further determine whether SOX1 had effect on apoptosis, we performed flow cytometry using annexin V-APC/7-AAD to examine the apoptosis rate of CCA cells. However, as was shown in Fig. 2F, overexpression of SOX1 had no effect on apoptosis in CCA cells. Overall, these results suggested that SOX1 inhibited the proliferation of CCA cells in vitro and tumor growth in vivo.

### MicroRNA-155-5p directly targets 3′UTR of SOX1 and inhibits expression of SOX1, and is overexpression in CCA tissues

**Fig. 3:**
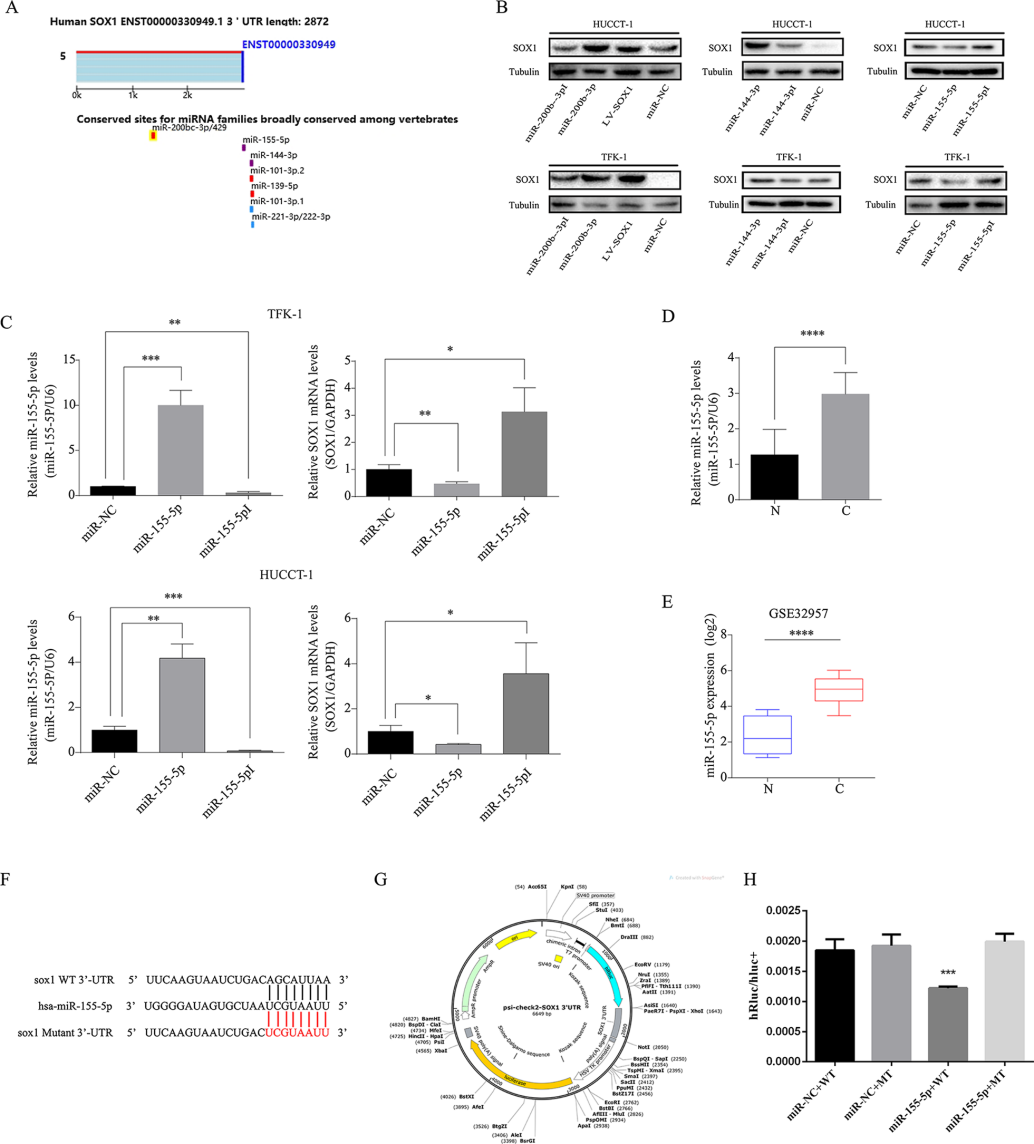
MicroRNA-155-5p directly targets 3′UTR of SOX1 and inhibits expression of SOX1, and is overexpression in CCA tissues. **A** Targetscan was used to screen candidate miRNAs regulating SOX1. **B** SOX1 protein levels in CCA cells with overexpression or inhibition of candidate miRNAs. **C** The relative mRNA level of SOX1 and miR-155-5p were determined respectively in TFK-1 and HUCCT-1 cells transfected with miR-NC, miR-155-5p and miR-155-5pI by RT-PCR. *p  < 0.05, **p  < 0.01, *** p  < 0.001. **D** The relative levels of miR-155-5p expression in four normal bile duct tissues and nine CCA tissues was analysis by RT-PCR. ****p  < 0.0001. **E** Box plots of miR-155-5p expression in CCA tissues compared to normal bile duct tissues. Data sourced from GEO databases GSE32957. ****p  < 0.0001. **F** Schematic illustration of the potential biding motifs for miR-155-5p in the wild-type (WT) 3′-UTR of SOX1 and their mutant-type (MT). **G** The plasmid map of psiCHECK2 which contained a SOX1 3′UTR-WT predicted binding site. **H** Relative activity of luciferase reporters with SOX1 3′UTR-WT and SOX1 3′UTR-MT after co-transfection with miR-155-5p mimics in HEK-293 T cells. ***p  < 0.001

To elucidate the mechanisms underlying the regulation of SOX1, we focused on microRNA, which has been proven to play an important role in a variety of tumors. One of the classic mechanisms of miRNA is via their specific binding to the 3′UTR of the target gene, which inhibits the translation and reduces the stability of the target mRNA, leading to decreased expression of the target protein [19]. We used Targetscan (http://www.targetscan.org/vert_71/) to screen out a variety of candidate miRNAs and chose the top three miRNA (miR-200b-3p, miR-144-3p, miR-155-5p) as candidates (Fig. 3A). Then we transfected the mimic and inhibitor of these candidate miRNAs to TFK-1 and HUCCT-1 and detected the expression of SOX1. The results of Western blot showed that neither the miR-200b-3p nor the miR-144-3p reduced the expression level of SOX1. On the other hand, only miR-155-5p significantly reduced the expression of SOX1, which was confirmed by western blot and qPCR (Fig. 3B, C). The quantification of western blots of Fig. 3B is showed in Additional file 1: Figure. S1B.  Further RT-PCR of 9 primary CCAs and 4 normal bile duct tissues indicated that CCA samples had higher levels of miR-155-5p expression (Fig. 3D). The expression of miR-155-5p in CCA patients based on data from the public GEO database (GSE32957) also showed the same results (Fig. 3E). Subsequently, we found that miR-155-5p may bind to the 3′UTR site of SOX1 (Fig. 3F). We introduced the wild-type (WT) or mutant-type (MT) 3′UTR of SOX1 into psiCHECK2 plasmids (Fig. 3G). The luciferase activity assay suggested that the mimic of miR-155-5p successfully decreased the luciferase activity when it was added to the WT; however, the luciferase activity of mimic which was added to MT showed no obvious change (Fig. 3H). Overall, these results showed that miR-155-5p had higher level in CCA tissues, and it suppressed the expression of SOX1 through binding to its 3′UTR region.

### MiR-155-5p inhibits SOX1 to activate the RAF/MEK/ERK pathway

**Fig. 4:**
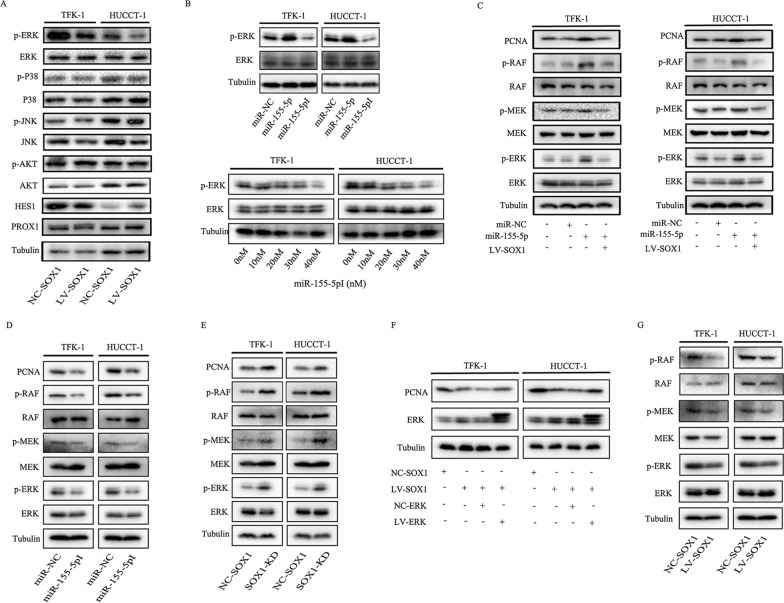
Mir-155-5p inhibits SOX1 leading to activation of the Raf/MEK/ERK pathway. **A** Cells were transfected with lentiviral negative control vector (NC-SOX1) or lentiviral SOX1 (LV-SOX1) for 72 h. Protein expressions of SOX1, HES1, PROX1, p-AKT, p-JNK, and p-P38 were examined by Western blot. **B** Above panel: protein levels of ERK and p-ERK in HUCCT-1 and TFK-1 cells transfected with miR-negative control (miR-NC), miR-155-5p-mimic (miR-155-5p), and miR-155-5p-inhibitor (miR-155-5pI). Below panel: TFK-1 and HUCCT-1 cells was treated with different concentrations of miR-155-5pI. The protein level of ERK and p-ERK were detected by Western blot. **C** Protein levels of central members of MAPK/ERK signaling (RAF, p-RAF, MEK, p-MEK, ERK and p-ERK) and downstream of ERK (PCNA) in TFK-1 and HUCCT-1 cells were detected by Western blot. **D** TFK-1 and HUCCT-1 cells was transfected with miR-negative control (miR-NC) and miR-15-5p inhibitor (miR-155-5pI), then protein levels of central members of MAPK/ERK signaling (RAF, p-RAF, MEK, p-MEK, ERK and p-ERK) and PCNA were detected by Western blot. **E** TFK-1 and HUCCT-1 cells was transfected with lenvisual carrying SOX1-RNAi (SOX1-KD), then protein levels of central members of MAPK/ERK signaling (RAF, p-RAF, MEK, p-MEK, ERK and p-ERK) and PCNA were detected by Western blot. **F** Cells were infected with NC-SOX1, LV-SOX1, LV-SOX1  +  NC-ERK and LV-SOX1  +  LV-ERK, and then detect changes in ERK and PCNA protein levels by western blot. **G** Protein levels of central members of MAPK/ERK signaling (RAF, p-RAF, MEK, p-MEK, ERK and p-ERK) was detected in xenograft tumor samples which had been transfected with NC-SOX1 and LV-SOX1

The above experimental results suggested that SOX1 can inhibit the proliferation of CCA cells. To further explore the underlying molecular mechanisms, we screened the downstream signaling pathway of SOX1. In a study by Guan et al. [7] SOX1 was shown to interact with β-Catenin in the Wnt signaling pathway and reverse the malignant biological behavior of nasopharyngeal carcinoma by inhibiting the activation of β-Catenin. SOX1 was also found to interact with *hes1*, which is the downstream target gene of notch1. Interestingly, Zhou et al. [20] reported that TRIP13 promotes the proliferation and invasion of epithelial ovarian cancer through Notch signaling pathway. Liao et al. [21] found that hepatitis B virus X protein can activate the Notch pathway, thus promoting the development of liver cancer. In the study by Li et al. [22] miR-1179 was found to inhibit the metastasis of breast cancer cells by inhibiting Notch signaling pathway and Hes1. In addition, SOX1 can block cell cycle exit by Prox1, which induces neuronal differentiation in vitro and in vivo [23]. Hogstrom et al. suggested that Prox1 might play a role by inhibiting Notch signaling pathway in colon cancer [24]. To further explore the mechanisms of SOX1 in CCA, we detected the expression of two key proteins that interact with SOX1 (HES1, PROX1) and the signaling pathways related to cholangiocarcinoma (AKT, JNK, P38, ERK) by Western blot. Interestingly, only the phosphorylation level of ERK changed significantly, whereas the expression level of β-catenin, PROX1, HES1 and the phosphorylation level of AKT, JNK, P38 remained stable. These observations suggested that overexpression of SOX1 decreased the phosphorylation level of ERK, while there was no significant change in total ERK expression (Fig. 4A).

The above experiments showed that miR-155-5p suppressed the expression of SOX1, and that SOX1 suppresses CCA via the ERK pathway. Therefore, we hypothesized that inhibition of SOX1 by miR-155-5p also promotes CCA via ERK signaling pathway. To address it, we transfected CCA cells with miR-155-5p and miR-155-5p inhibitor (miR-155-5pI). The results suggested that miR-155-5p activated the ERK pathway by increasing the phosphorylation of ERK in HUCCT-1, but miR-155-5pI may suppress the ERK pathway in a concentration-dependent manner (Fig. 4B).

ERK signaling pathway is known to be one of the mitogen-activated protein kinase (MAPK) signaling pathways. Phosphorylation can lead to activation of ERK, which can promote cancer cell proliferation, invasion, and metastasis. Downstream of KRAS, the RAF → MEK → ERK signaling pathway plays a central role in carcinogenesis [25]. It is well established that, once activated, the MAPKs phosphorylate their respective substrates thereby controlling proliferation [26], and one of the classical ways that promote proliferation is activation of Proliferating Cell Nuclear Antigen (PCNA) [27]. We found the same results in Kyoto Encyclopedia of Genes and Genomes (KEGG). As we know, PCNA serves as an excellent marker of proliferating cells, so we think PCNA is a potential target of the RAF/MEK/ERK pathway. We next investigated the potential activation by miR-155-5p of the RAF/MEK/ERK and PCNA. Western blot showed that miR-155-5p also enhanced the phosphorylation of RAF/MEK/ERK, then activated PCNA, while miR-155-5pI presented the completely opposite effect. Importantly, reintroduction of SOX1 reversed the phenomenon of high phosphorylation level of RAF/MEK/ERK induced by miR-155-5p, which suggests a potential action of miR-155-5p and SOX1 on proliferation of CCA cells (Fig. 4C, D). Furthermore, western blot showed SOX1-KD enhanced phosphorylation of RAF/MEK/ERK, which confirm the effect of SOX1 on RAF/MEK/ERK pathway (Fig. 4E). In order to determine whether SOX1 affects the PCNA via ERK, we therefore used lentiviruses to overexpress both SOX1 and ERK (LV-ERK) in CCA cells. Accordingly, a lower expression of PCNA was observed with LV-SOX1, but it reversed when overexpress ERK in these cells (Fig. 4F). All the quantification of western blots of Fig. 4 is showed in Additional file 2: Figure. S2 to Additional file 3: Figure. S3. This result established that SOX1 could inhibit PCNA via RAF/MEK/ERK.

To find out whether these hypotheses also holds on in vivo, expression of RAF/MEK/ERK were detect in xenograft tumor samples. Similarly, lower phosphorylation of RAF/MEK/ERK was found in LV-SOX1 (Fig. 4G), which confirmed the previous observation. These data indicated that miR-155-5p may suppress SOX1 to activate the RAF/MEK/ERK signaling pathway.

### MiR-155-5p promotes the proliferation of CCA cells via inhibiting the expression of SOX1

**Fig. 5:**
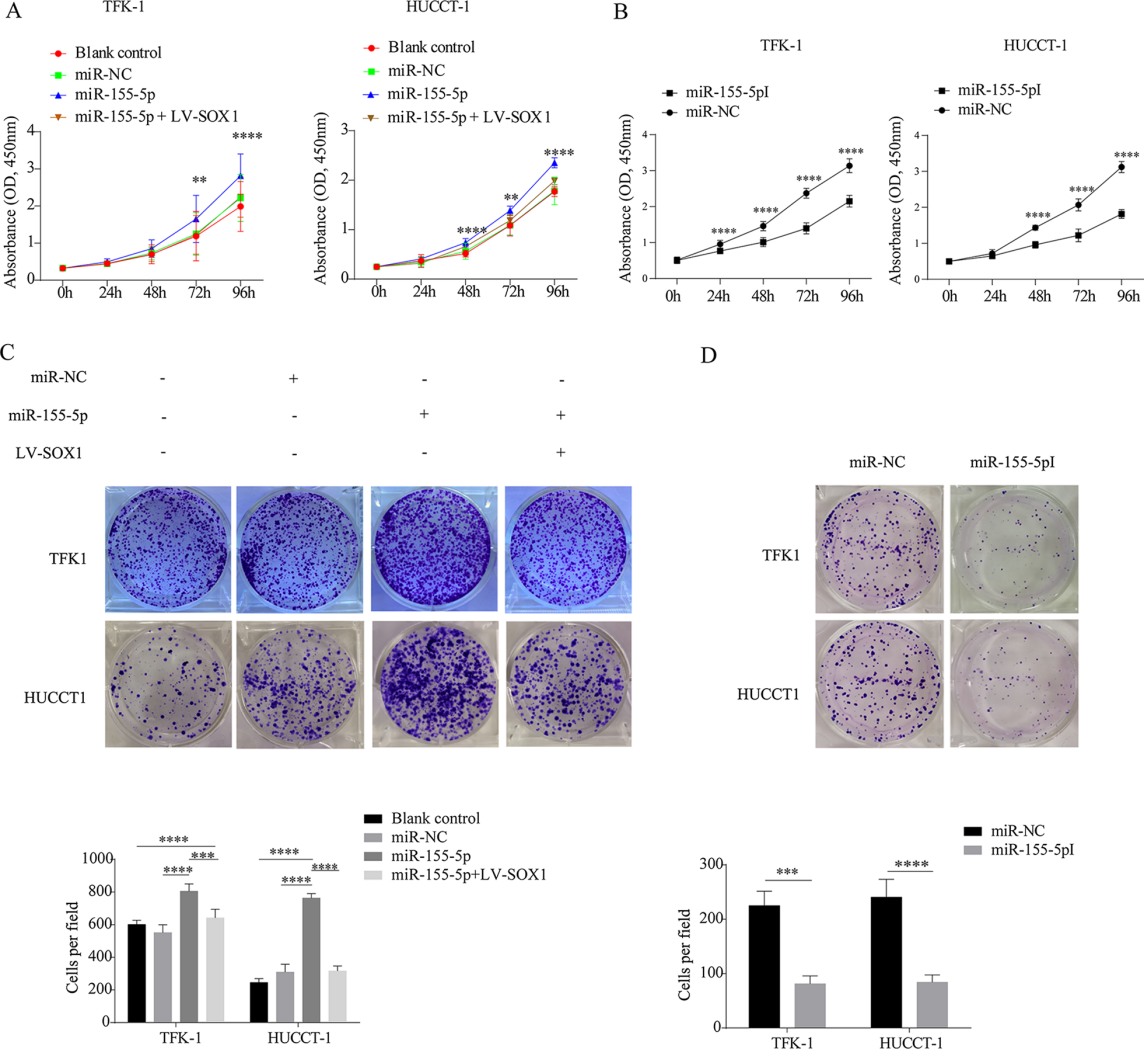
The miR-155-5p/SOX1 axis regulates the proliferation of CCA cells. **A**, **B** CCK-8 assay was performed to compare cell proliferation in blank control, miR-NC, miR-155-5p, miR-155-5p  +  SOX1 and miR-155-5pI in TFK-1 and HUCCT-1 cells. All experiments were performed in triplicate and data are presented as mean  ±  standard deviation. **p  < 0.01, ****p  < 0.0001. **C**, **D** Representative images of colony formation assays of TFK-1 and HUCCT-1 cells in groups of blank control, miR-NC, miR-155-5p, miR-155-5p  +  SOX1 and miR-155-5pI. ***p  < 0.001, ****p  < 0.0001

To study the role of miR-155-5p in CCA cells, we performed CCK-8 and plate clone assay. As expected, miR-155-5p promoted the proliferation of CCA cells; however, overexpression of SOX1 blocked the stimulatory effect of miR-155-5p on the proliferation of CCA cells, indicating that downregulation of SOX1 is an important mechanism by which miR-155-5p promotes cell proliferation (Fig. 5A, C). When inhibit the expression of miR-155-5p, the proliferation of CCA cells and the number of clones had decreased (Fig. 5B, D). These data indicated that miR-155-5p may promote the proliferation of CCA cells via inhibiting the expression of SOX1.

## Discussion

**Fig. 6:**
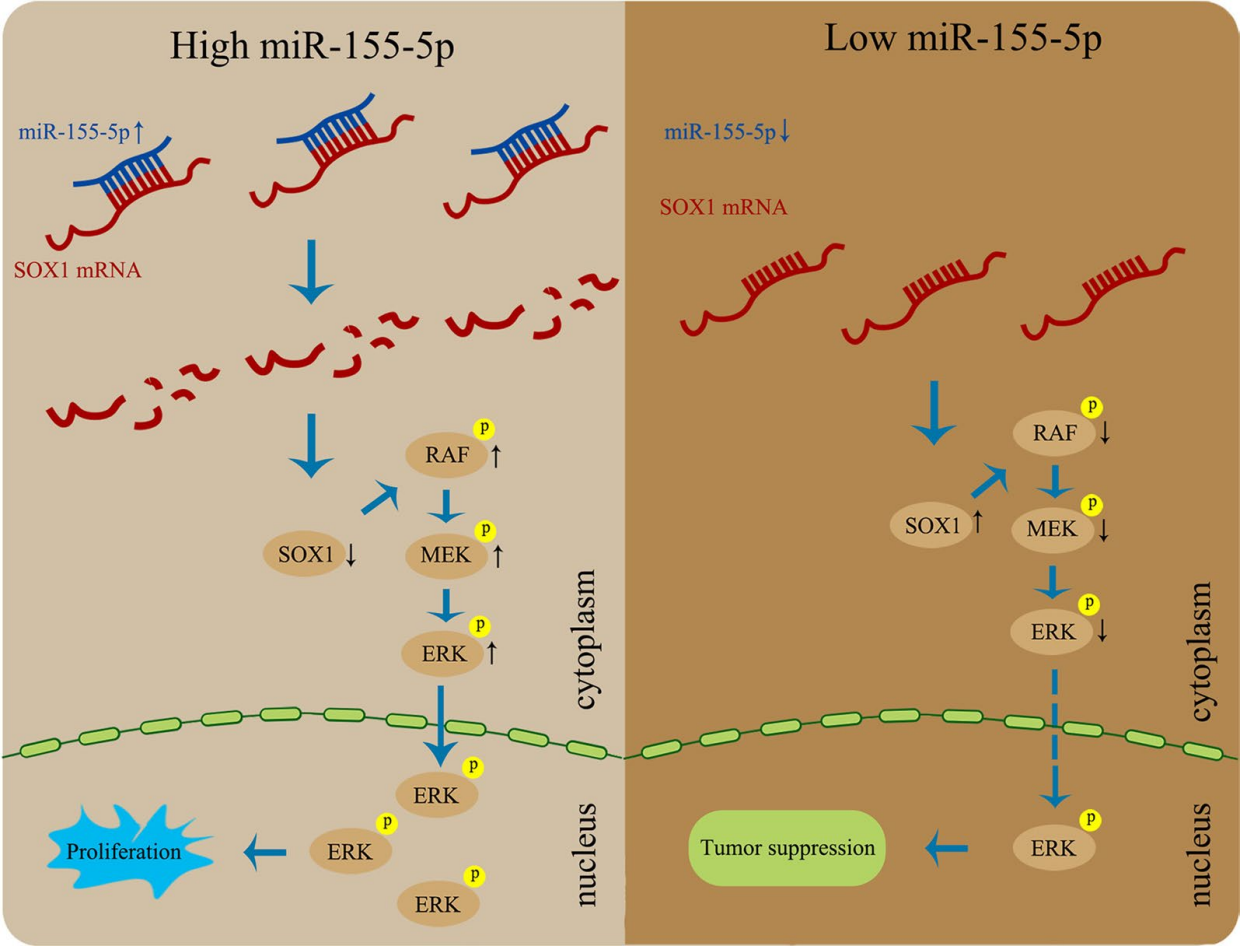
A Schematic illustration of the mechanism by which Mir-155-5p promotes progression of CCA. Mir-155-5p represses SOX1 expression by binding to the 3′UTR region of SOX1, which activates the Raf/MEK/ERK pathway, thus promoting CCA progression. However, low expression level of miR-155-5p would not lead to inhibition of SOX1; in this setting, SOX1 would suppress the Raf/MEK/ERK pathway and inhibit CCA progression

SOX1 is a member of the transcription factor sox family, which plays an important role in embryo and postnatal development. Studies have also shown an inhibitory effect of SOX1 on a variety of tumors. In addition, many studies have shown that SOX1 can be used as a biomarker for a variety of tumors, such as cervical cancer, colon cancer, glioma, liver cancer, ovarian cancer, and small cell lung cancer [28–33]. However, to the best of our knowledge, the association between SOX1 and CCA has not been reported. In this study, we first demonstrated the downregulation of SOX1 in CCA tissues. Then, we confirmed that SOX1 significantly suppressed CCA cell proliferation and growth, in vitro and in vivo. In addition, SOX1 may be regulated by miR-155-5p. Finally, we confirmed that miR-155-5p/SOX1/RAF/MEK/ERK axis may play an important role in CCA development (Fig. 6).

To understand the role of SOX1 in CCA, we first examined the expression of SOX1 in CCA clinical specimens and the data on the public GEO database (GSE32225, GSE76297). The results showed downregulation of SOX1 in CCA tissues. Through explore the clinicopathologic characteristics of patient, we found that low level of SOX1 was associated with large tumor size (P  < 0.05), which indicate that SOX1 may associated with proliferation of tumor. We used lentiviruses to alter the expression of SOX1 in CCA cells, and assessed the effects using CCK-8 assay, plate clone assay, flow cytometry and in subcutaneous tumor-bearing mice. The results showed that SOX1 significantly suppressed the proliferation of CCA cells, both in vitro and in vivo. These data indicated that SOX1 inhibited the biological behavior of CCA.

An increasing number of studies have demonstrated the crucial role of miRNAs in cancer cell proliferation. To explore the upstream factors that regulate SOX1 expression in CCA, we focused on microRNA. Using bioinformatics analysis, we screened out a variety of candidate miRNAs and selected the top three miRNA (miR-200b-3p, miR-144-3p, miR-155-5p) as candidates. Subsequently, Western blot assay, qPCR and luciferase activity assay were performed to assess our hypothesis. The observations showed that miR-155-5p may decrease the expression of SOX1 by binding to its 3’UTR region. Furthermore, qPCR of clinical samples of CCA and bioinformatics analysis data from the public GEO database (GSE32957) indicated that CCA tissues had higher expression levels of miR-155-5p compared with normal bile ducts, which confirmed the important role of miR-155-5p in CCA.

The potential molecular mechanism of the association of SOX1 with CCA was also investigated in this research. Previous studies have shown that ERK can be activated to promote the development of colon cancer [34]. CD110 has been shown to promote the progression of pancreatic cancer through activating the ERK [35]. 1,25-(OH)_2_D_3_ was found to inhibit the proliferation, invasion, and metastasis of breast cancer cells by inhibiting the activation of ERK signaling pathway [36]. Thus, ERK expression is critical for tumor development and its hyperactivation plays a major role in cancer development and progression. The RAF/MEK/ERK pathway is the most important signaling cascade among all MAPK signal transduction pathways, and plays a crucial role in the survival and development of tumor cells. Ras can bind and translocate RAF from the cytoplasm to the cell membrane, where RAF is activated. Activated RAF-1 continues to activate downstream MEK and ERK, and then regulates the downstream gene expression, such as PCNA [37]. PCNA is an essential factor in DNA replication and repair, and is involved in cell survival [38], which can be activated by MAPK/ERK pathway [27]. The expression level of PCNA may reflect the proliferative ability of the CCA cells. Interestingly, we found that overexpression of SOX1 in CCA cells decreased the phosphorylation of ERK. Then we explored the relationship between miR-155-5p, SOX1 and the RAF/MEK/ERK pathway. We observed that overexpression of miR-155-5p activated the RAF/MEK/ERK pathway by enhancing the phosphorylation of RAF/MEK/ERK, then further improve the expression level of PCNA, leading to increased proliferation of CCA cells. However, upregulation of SOX1 expression in cells with overexpression of miR-155-5p significantly decreased the activation of the pathway, as well as PCNA, leading to restoration of CCA cell proliferation. When we enhanced expression of ERK in cells with overexpression of SOX1, PCNA increase again. In addition, inhibit the expression of miR-155-5p induced the decrease of phosphorylation level of RAF/MEK/ERK and suppressed the proliferation of CCA cells. Similarly, western blot of xenograft tumor samples showed the same results. In CCA cells, knockdown SOX1 leading to a decrease phosphorylation of RAF/MEK/ERK. These data indicated that miR-155-5p promoted CCA through its effect on SOX1 leading to activation of the RAF/MEK/ERK signaling pathway.

## Conclusions

In summary, we found that SOX1 is downregulated expression in CCA patients, it suppressed the RAF/MEK/ERK pathway by decreasing the phosphorylation of RAF, MEK, and ERK, and inhibited the proliferation of CCA cells in vitro and suppressed tumor growth in vivo. MiR-155-5p, which was upregulated in CCA patients, decreasing the expression of SOX1 by binding to its 3′UTR, which activated the RAF/MEK/ERK signaling pathway and promoted CCA progression. Our findings demonstrate the critical role of miR-155-5p/SOX1/RAF/MEK/ERK axis in CCA progression, which may provide a novel therapeutic target for CCA.

## Supplementary Information

**Additional file 1: Figure S1.** Quantification of western blots of Figs. 2E,  3B, corresponding to A, B respectively.

**Additional file 2: Figure S2.** Quantification of western blots of Fig. 4A–C, corresponding to A, B, C respectively.

**Additional file 3: Figure S3.** Quantification of western blots of Fig. 4D–G, corresponding to A, B, C, D respectively.

## Data Availability

The datasets used and/or analyzed during the current study are available from the corresponding author on reasonable request. All data have been submitted as supplemental material to editors.

## Abbreviations

CCA: Cholangiocarcinoma
SOX1: SRY-related HMG-box1
GEO: Gene expression omnibus
miRNA: MicroRNA
WT: Wild type
MT: Mutant type

## References

[1] Xie Y, Wang Y, Li J, Hang Y, Jaramillo L, Wehrkamp CJ (2018). Cholangiocarcinoma therapy with nanoparticles that combine downregulation of MicroRNA-210 with inhibition of cancer cell invasiveness. Theranostics.

[2] Rizvi S, Khan SA, Hallemeier CL, Kelley RK, Gores GJ (2018). Cholangiocarcinoma—evolving concepts and therapeutic strategies. Nat Rev Clin Oncol.

[3] Radtke A, Konigsrainer A (2016). Surgical therapy of cholangiocarcinoma. Visc Med.

[4] Wang P, Song X, Utpatel K, Shang R, Yang YM, Xu M (2019). MEK inhibition suppresses K-Ras wild-type cholangiocarcinoma in vitro and in vivo via inhibiting cell proliferation and modulating tumor microenvironment. Cell Death Dis.

[5] Doherty B, Nambudiri VE, Palmer WC (2017). Update on the diagnosis and treatment of cholangiocarcinoma. Curr Gastroenterol Rep.

[6] Kamachi Y, Kondoh H (2013). Sox proteins: regulators of cell fate specification and differentiation. Development.

[7] Guan Z, Zhang J, Wang J, Wang H, Zheng F, Peng J (2014). SOX1 down-regulates beta-catenin and reverses malignant phenotype in nasopharyngeal carcinoma. Mol Cancer.

[8] Kan L, Israsena N, Zhang Z, Hu M, Zhao LR, Jalali A (2004). Sox1 acts through multiple independent pathways to promote neurogenesis. Dev Biol.

[9] Lazarus KA, Hadi F, Zambon E, Bach K, Santolla MF, Watson JK (2018). BCL11A interacts with SOX2 to control the expression of epigenetic regulators in lung squamous carcinoma. Nat Commun.

[10] Xiao Y, Sun Y, Liu G, Zhao J, Gao Y, Yeh S (2019). Androgen receptor (AR)/miR-520f-3p/SOX9 signaling is involved in altering hepatocellular carcinoma (HCC) cell sensitivity to the Sorafenib therapy under hypoxia via increasing cancer stem cells phenotype. Cancer Lett.

[11] Zhang Y, Jiang F, Bao W, Zhang H, He X, Wang H (2016). SOX17 increases the cisplatin sensitivity of an endometrial cancer cell line. Cancer Cell Int.

[12] Tsao CM, Yan MD, Shih YL, Yu PN, Kuo CC, Lin WC (2012). SOX1 functions as a tumor suppressor by antagonizing the WNT/beta-catenin signaling pathway in hepatocellular carcinoma. Hepatology.

[13] Rad A, Esmaeili Dizghandi S, Abbaszadegan MR, Taghechian N, Najafi M, Forghanifard MM (2016). SOX1 is correlated to stemness state regulator SALL4 through progression and invasiveness of esophageal squamous cell carcinoma. Gene.

[14] Li N, Li S (2015). Epigenetic inactivation of SOX1 promotes cell migration in lung cancer. Tumour Biol.

[15] Qi Y, Wang D, Huang W, Wang B, Huang D, Xiong F (2019). CyclinD1 inhibits dicer and crucial miRNA expression by chromatin modification to promote the progression of intrahepatic cholangiocarcinoma. J Exp Clin Cancer Res.

[16] Sia D, Hoshida Y, Villanueva A, Roayaie S, Ferrer J, Tabak B (2013). Integrative molecular analysis of intrahepatic cholangiocarcinoma reveals 2 classes that have different outcomes. Gastroenterology.

[17] Chaisaingmongkol J, Budhu A, Dang H, Rabibhadana S, Pupacdi B, Kwon SM (2017). Common molecular subtypes among Asian hepatocellular carcinoma and cholangiocarcinoma. Cancer Cell.

[18] Oishi N, Kumar MR, Roessler S, Ji J, Forgues M, Budhu A (2012). Transcriptomic profiling reveals hepatic stem-like gene signatures and interplay of miR-200c and epithelial-mesenchymal transition in intrahepatic cholangiocarcinoma. Hepatology.

[19] Ventura A, Jacks T (2009). MicroRNAs and cancer: short RNAs go a long way. Cell.

[20] Zhou XY, Shu XM (2019). TRIP13 promotes proliferation and invasion of epithelial ovarian cancer cells through Notch signaling pathway. Eur Rev Med Pharmacol Sci.

[21] Liao B, Zhou H, Liang H, Li C (2017). Regulation of ERK and AKT pathways by hepatitis B virus X protein via the Notch1 pathway in hepatocellular carcinoma. Int J Oncol.

[22] Li WJ, Xie XX, Bai J, Wang C, Zhao L, Jiang DQ (2018). Increased expression of miR-1179 inhibits breast cancer cell metastasis by modulating Notch signaling pathway and correlates with favorable prognosis. Eur Rev Med Pharmacol Sci.

[23] Elkouris M, Balaskas N, Poulou M, Politis PK, Panayiotou E, Malas S (2011). Sox1 maintains the undifferentiated state of cortical neural progenitor cells via the suppression of Prox1-mediated cell cycle exit and neurogenesis. Stem Cells.

[24] Elsir T, Smits A, Lindstrom MS, Nister M (2012). Transcription factor PROX1: its role in development and cancer. Cancer Metastasis Rev.

[25] Kinsey CG, Camolotto SA, Boespflug AM, Guillen KP, Foth M, Truong A (2019). Protective autophagy elicited by RAF→MEK→ERK inhibition suggests a treatment strategy for RAS-driven cancers. Nat Med.

[26] Chang L, Karin M (2001). Mammalian MAP kinase signalling cascades. Nature.

[27] Lausson S, Cressent M (2011). Signal transduction pathways mediating the effect of adrenomedullin on osteoblast survival. J Cell Biochem.

[28] Rogeri CD, Silveira HCS, Causin RL, Villa LL, Stein MD, de Carvalho AC (2018). Methylation of the hsa-miR-124, SOX1, TERT, and LMX1A genes as biomarkers for precursor lesions in cervical cancer. Gynecol Oncol.

[29] Huang J, Tan ZR, Yu J, Li H, Lv QL, Shao YY (2017). DNA hypermethylated status and gene expression of PAX1/SOX1 in patients with colorectal carcinoma. Onco Targets Ther.

[30] Garcia I, Aldaregia J, Marjanovic Vicentic J, Aldaz P, Moreno-Cugnon L, Torres-Bayona S (2017). Oncogenic activity of SOX1 in glioblastoma. Sci Rep.

[31] Liu XY, Fan YC, Gao S, Zhao J, Chen LY, Li F (2017). Methylation of SOX1 and VIM promoters in serum as potential biomarkers for hepatocellular carcinoma. Neoplasma.

[32] Lipka AF, Verschuuren JJ, Titulaer MJ (2012). SOX1 antibodies in Lambert-Eaton myasthenic syndrome and screening for small cell lung carcinoma. Ann NY Acad Sci.

[33] Kaur M, Singh A, Singh K, Gupta S, Sachan M (2016). Development of a multiplex MethyLight assay for the detection of DAPK1 and SOX1 methylation in epithelial ovarian cancer in a north Indian population. Genes Genet Syst.

[34] Wei F, Zhang T, Deng SC, Wei JC, Yang P, Wang Q (2019). PD-L1 promotes colorectal cancer stem cell expansion by activating HMGA1-dependent signaling pathways. Cancer Lett.

[35] Yan Z, Ohuchida K, Zheng B, Okumura T, Takesue S, Nakayama H (2019). CD110 promotes pancreatic cancer progression and its expression is correlated with poor prognosis. J Cancer Res Clin Oncol.

[36] Zheng W, Cao L, Ouyang L, Zhang Q, Duan B, Zhou W (2019). Anticancer activity of 1,25-(OH)2D3 against human breast cancer cell lines by targeting Ras/MEK/ERK pathway. Onco Targets Ther.

[37] Guo YJ, Pan WW, Liu SB, Shen ZF, Xu Y, Hu LL (2020). ERK/MAPK signalling pathway and tumorigenesis. Exp Ther Med.

[38] Cardano M, Tribioli C, Prosperi E (2020). Targeting proliferating cell nuclear antigen (PCNA) as an effective strategy to inhibit tumor cell proliferation. Curr Cancer Drug Targets.

