# Supplementary figures and images for: MiR-155-5p suppresses SOX1 to promote proliferation of cholangiocarcinoma via RAF/MEK/ERK pathway
Source: Cancer Cell Int. 2021 Dec 7;21:656. doi: 10.1186/s12935-021-02374-0 (PMC8650398; doi:10.1186/s12935-021-02374-0)

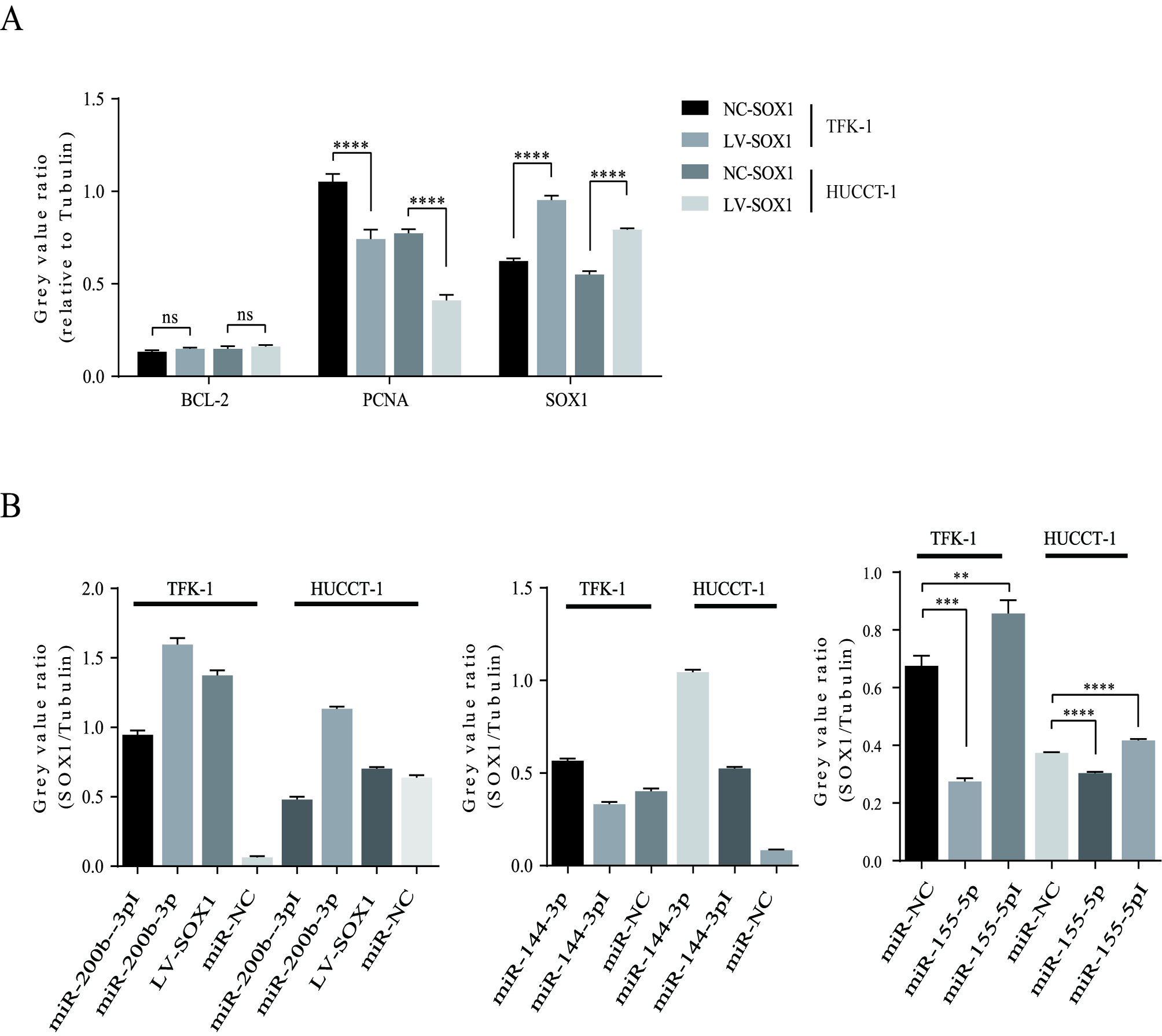

Supplement: Supplementary file 1 — Additional file 1: Figure S1. Quantification of western blots of Figs. 2E, 3B, corresponding to A, B respectively. [file 12935_2021_2374_MOESM1_ESM.tif]

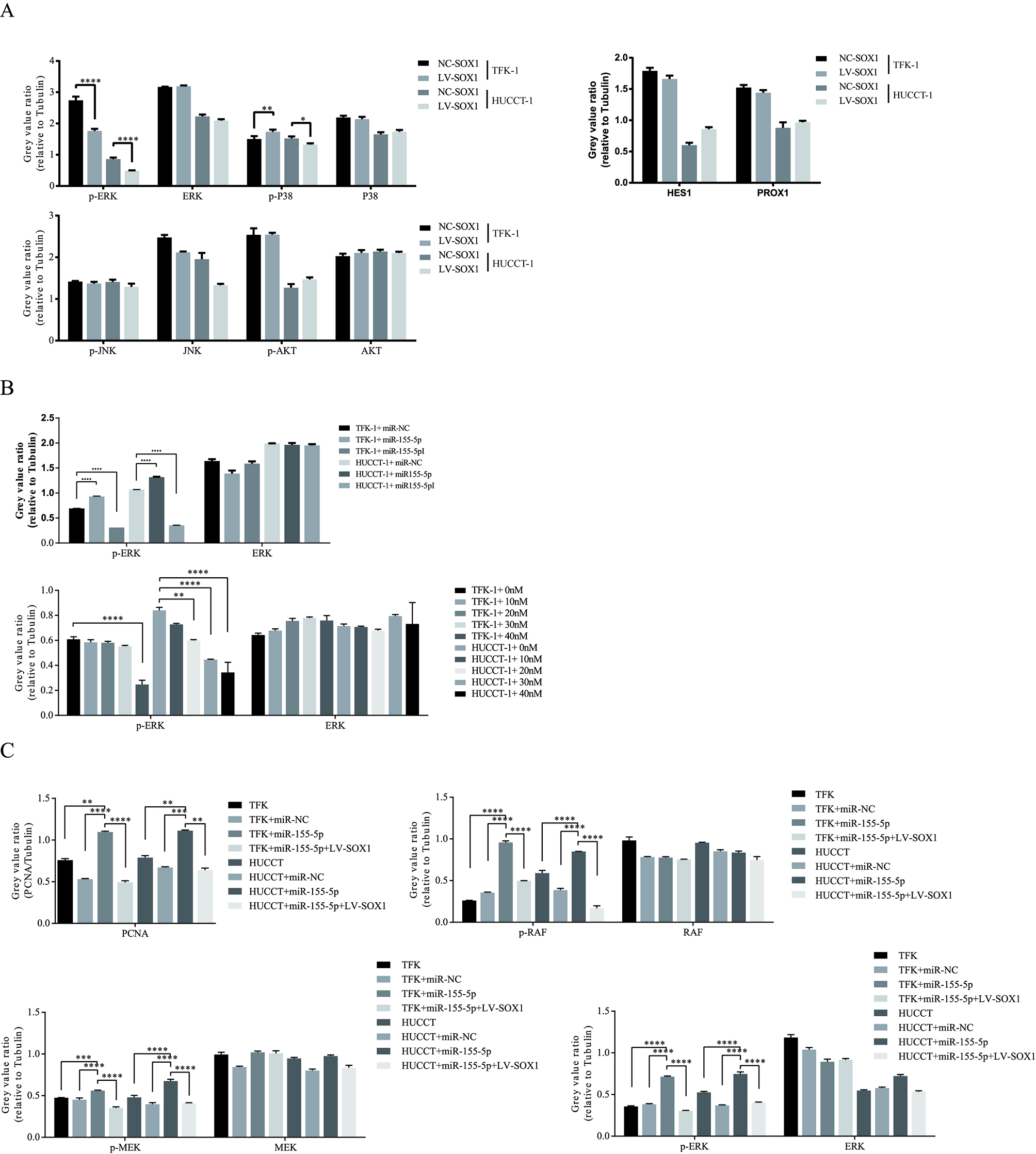

Supplement: Supplementary file 2 — Additional file 2: Figure S2. Quantification of western blots of Fig. 4A–C, corresponding to A, B, C respectively. [file 12935_2021_2374_MOESM2_ESM.tif]

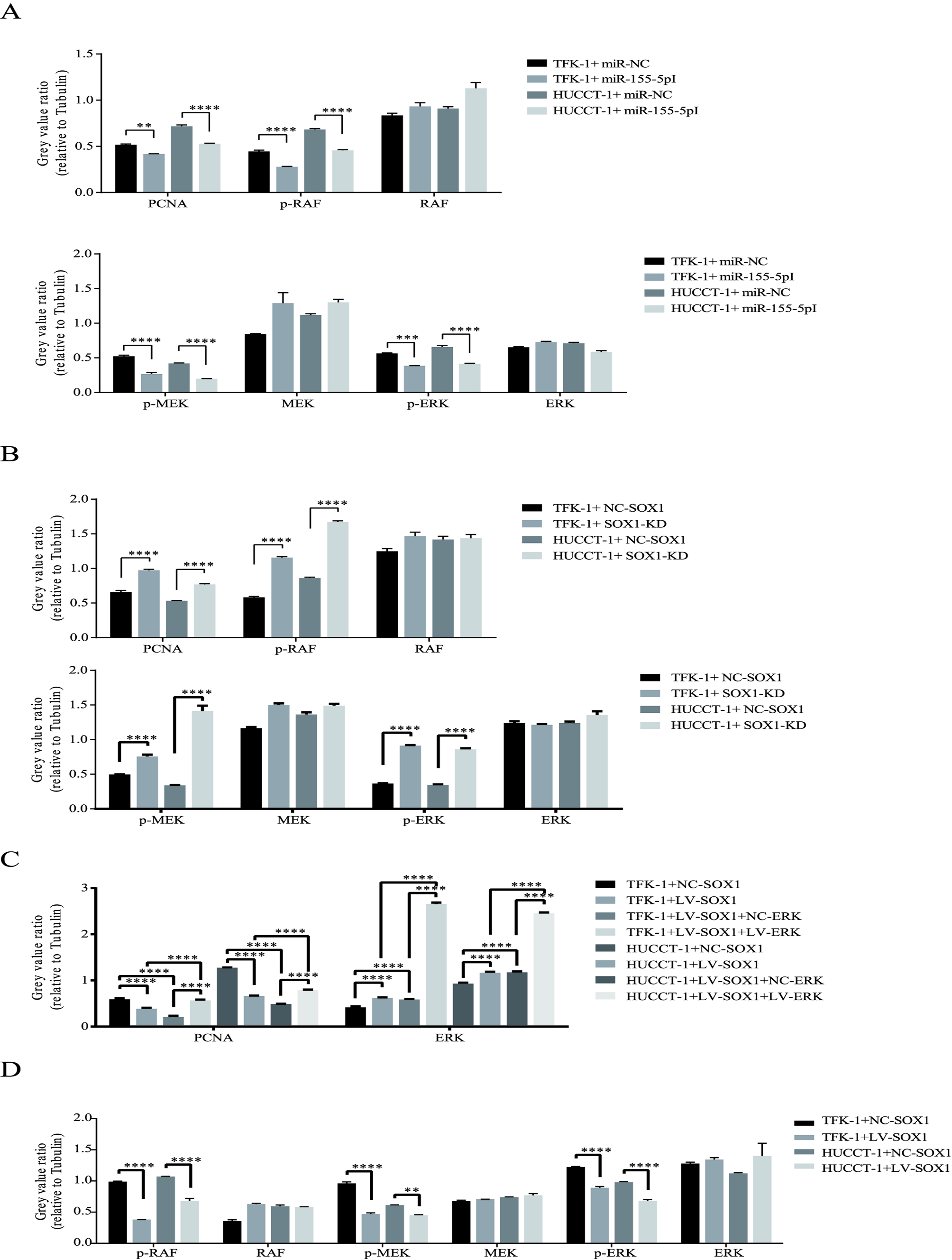

Supplement: Supplementary file 3 — Additional file 3: Figure S3. Quantification of western blots of Fig. 4D–G, corresponding to A, B, C, D respectively. [file 12935_2021_2374_MOESM3_ESM.tif]
